# Simplified Luminal Water Imaging for the Detection of Prostate Cancer From Multiecho T_2_ MR Images

**DOI:** 10.1002/jmri.26608

**Published:** 2018-12-19

**Authors:** William Devine, Francesco Giganti, Edward W. Johnston, Harbir S. Sidhu, Eleftheria Panagiotaki, Shonit Punwani, Daniel C. Alexander, David Atkinson

**Affiliations:** ^1^ Centre for Medical Imaging University College London London United Kingdom; ^2^ Department of Radiology University College London Hospital NHS Foundation Trust London United Kingdom; ^3^ Division of Surgery and Interventional Science University College London London United Kingdom; ^4^ Centre for Medical Image Computing, Department of Computer Science University College London London United Kingdom

**Keywords:** microstructure, modeling, multiecho T2, prostate, cancer

## Abstract

**Background:**

Luminal water imaging (LWI) suffers less from imaging artifacts than the diffusion‐weighted imaging used in multiparametric MRI of the prostate. LWI obtains multicompartment tissue information from a multiecho T_2_ dataset.

**Purpose:**

To compare a simplified LWI technique with apparent diffusion coefficient (ADC) in classifying lesions based on groupings of PI‐RADS v2 scores. Secondary aims were to investigate whether LWI differentiates between histologically confirmed tumor and normal tissue as effectively as ADC, and whether LWI is correlated with the multicompartment parameters of the vascular, extracellular, and restricted diffusion for cytometry in tumors (VERDICT) diffusion model.

**Study Type:**

A subset of a larger prospective study.

**Population:**

In all, 65 male patients aged 49–79 were scanned.

**Field Strength/Sequence:**

A 32‐echo T_2_ and a six b‐value diffusion sequence (0, 90, 500, 1500, 2000, 3000 s/mm^2^) at 3T.

**Assessment:**

Regions of interest were placed by a board‐certified radiologist in areas of lesion and benign tissue and given PI‐RADS v2 scores.

**Statistical Tests:**

Receiver operating characteristic and logistic regression analyses were performed.

**Results:**

LWI classifies tissue as PI‐RADS 1,2 or PI‐RADS 3,4,5 with an area under curve (AUC) value of 0.779, compared with 0.764 for ADC. LWI differentiated histologically confirmed malignant from nonmalignant tissue with AUC, sensitivity, and specificity values of 0.81, 75%, and 87%, compared with 0.75, 83%, and 67% for ADC. The microstructural basis of the LWI technique is further suggested by the correspondence with the VERDICT diffusion‐based microstructural imaging technique, with α, *A*_*1*_ *, A*_*2*_, and LWF showing significant correlations.

**Data Conclusion:**

LWI alone can predict PI‐RADS v2 score groupings and detect histologically confirmed tumors with an ability similar to ADC alone without the limitations of diffusion‐weighted MRI. This is important, given that ADC has an advantage in these tests as it already informs PI‐RADS v2 scoring. LWI also provides multicompartment information that has an explicit biophysical interpretation, unlike ADC.

**Level of Evidence**: 3

**Technical Efficacy**: Stage 2

J. Magn. Reson. Imaging 2019;50:910–917.

PROSTATE CANCER (PCa) is the second most common cancer in the UK, accounting for 13% of all new cases, and the second most common cause of cancer death in males.^1^, ^2^, ^3^, ^4^ The suspicion of PCa is typically assessed using multiparametric magnetic resonance imaging (mp‐MRI) images, including T_2_‐weighted imaging (T_2_WI), diffusion‐weighted imaging (DWI), and dynamic contrast‐enhanced (DCE) measurements,^5^ which are then scored using an ordinal scale, most commonly the Prostate Imaging Reporting and Data System (PI‐RADS) v2 scheme.^6^, ^7^ mp‐MRI provides sensitivity and specificity values of between 87–93% and 41–47%, respectively, in the detection of clinically significant prostate cancer when used on a 1.5 T scanner.^8^

Current limitations of mp‐MRI are that its specificity when detecting tumors is low^8^ and there is only a moderate interreader agreement across all lesions (55–65%).^9^ PI‐RADS v2.0 on a 1.5 T scanner also classifies 35.6–44.7% of lesions as indeterminate,^8^ meaning that the method cannot distinguish whether a large number of cases are clinically significant prostate cancer or not, although in practice the classification may vary depending on the radiologist, the patient population, and the image quality. Furthermore, echo planar imaging (EPI)‐based diffusion scans often suffer from distortion, signal pile‐up, or stretching artifacts,^10^ leading to less accurate results and in some circumstances unusable images. Different studies have tried to solve some of these issues, particularly with regard to reducing the proportion of lesions classified as indeterminate,^11^, ^12^ but none has conclusively solved them.

The tissue within the prostate has three major components: luminal space, epithelial cells, and stromal cells, as shown in Fig. 1. The luminal space stores the fluid produced by the surrounding epithelial cells and the stromal cells form a matrix surrounding these compartments, giving the prostate structure and forcing the fluid out of the prostate during ejaculation. The luminal water imaging (LWI) technique models two compartments, one the luminal space with a distribution of long T_2_ values and the other both the stromal and epithelial cells with a distribution of short T_2_ values. The vascular, extracellular, and restricted diffusion for cytometry in tumors (VERDICT) technique models three compartments: the intracellular compartment, which represents the restricted diffusion of the epithelial cells, the extracellular‐extravascular compartment, which represents hindered diffusion within the luminal space and stroma, and the vascular compartment. The diffusivity values for the intracellular and extracellular‐extravascular compartments are assumed to be the same and of a lower value than the pseudo‐diffusivity of the vascular compartment.

**Figure 1:**
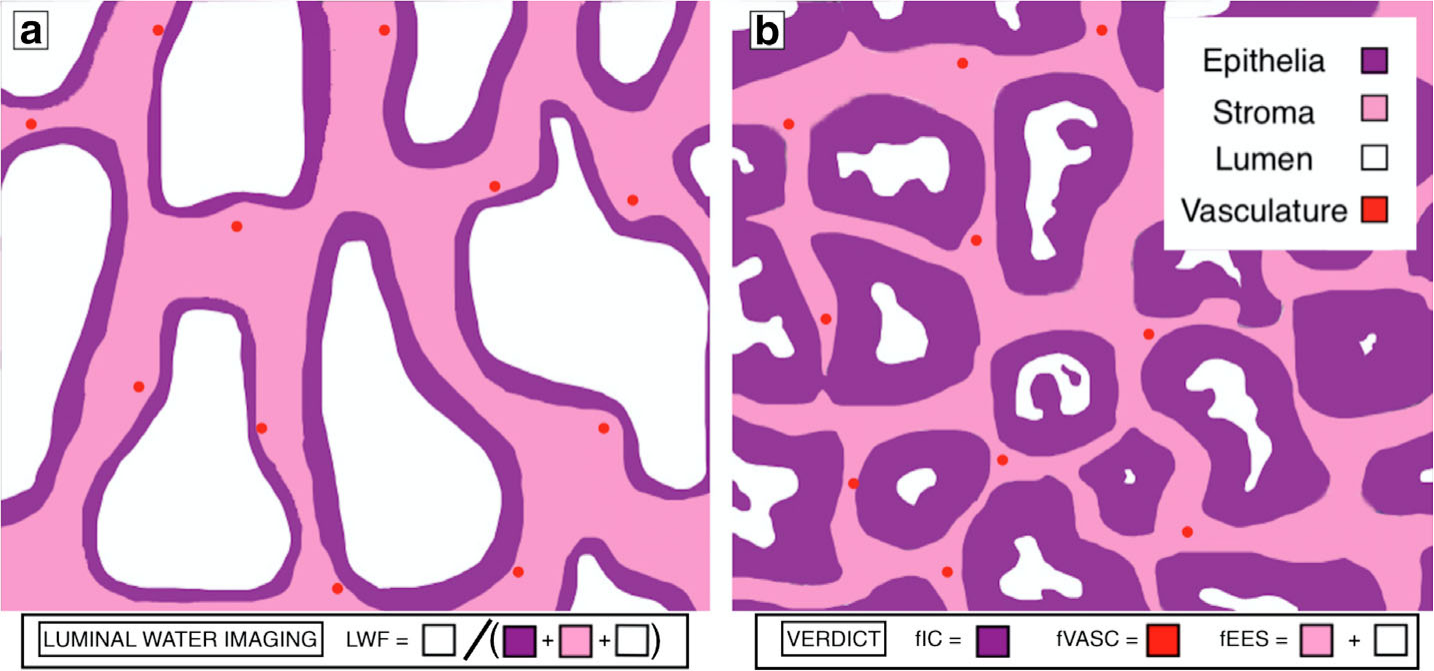
Diagram of prostate histology in **(a)** benign tissue **(b)** malignant adenocarcinoma. The four separate microenvironments present in the prostate are shown in the key. Using LWI, the short‐T_2_ component is made up of the stroma and epithelia and the long‐T_2_ component is made up of the lumen (the T_2_ of the vasculature is not considered to have a significant effect on the LWI model). Using the VERDICT model, the intracellular compartment is made up of epithelia, the extracellular‐extravascular compartment is made up of stroma, and lumen and the vascular compartment is made up of the vasculature.

The prostate is made up of a number of separate tissue zones. Each is made up of different proportions of gland, stroma, and epithelia. The central zone consists of large, irregularly shaped glands, cuboidal epithelial cells, and compact stromal tissue. The peripheral zone contains small, regularly arranged glands lined by columnar epithelial cells and surrounded by loosely interwoven stroma. The transition zone, composed of two lobules either side of the urethra, closely resembles the peripheral zone tissue.

Storas et al^13^ found that a multiecho T_2_ (ME‐T_2_) sequence is consistently better at probing tissue microstructure than a single echo T_2_ sequence, showing that in the prostate a mono‐exponential fit is only adequate for describing the underlying tissue in 10% of the subjects. Sabouri et al^14^ implemented a method to investigate quantitative T_2_ imaging in the prostate, proposing the LWI technique, which produces an estimate of the fractional volume of luminal water in each voxel of the prostate, the luminal water fraction (LWF). Sabouri et al^14^ have shown that there is a good correlation between LWF and histologically measured luminal fractional volume and determined that LWI shows promise in being able to detect PCa and predict Gleason score.^15^ However, the fitting method used has a high number of degrees of freedom, potentially making it vulnerable to noise and local minima. They used an echo train with 64 echoes, which is not available on all scanners. In addition, compared with using fewer echoes, a 64 echo train may be more vulnerable to the cumulative effects of imperfect refocusing pulses and have a higher specific absorption rate.

## Materials and Methods

### *Simulation*

A simulation was used to compare alternative methods for fitting the LWF. Signals from a tissue with a distribution of T_2_ values were simulated. Datasets typical of both acquisition schemes were used (Sabouri et al: echo time [TE] = 25 msec, number of echoes [NE] = 64; this study: TE = 31.25 msec, NE = 32) and two separate datasets were created on different assumptions about the underlying T_2_ distribution, one using two Gaussian peaks and the other using two delta peaks. The values used for the shorter T_2_ compartment were 20, 50, and 80 msec and for the longer T_2_ compartment were 300, 550, and 800 msec. Using a value of signal‐to‐noise ratio (SNR) of 100 and for ground truth LWF values of 0, 0.1, and 0.2 (a range typical of both tumor and normal tissue,^14^) the LWF was calculated from fits to each set of conditions.

### *Patient Selection*

In vivo data were acquired on a subcohort of 65 patients from a larger prospective study.^17^ The study received UK Research Ethics Committee approval on December 23, 2015. Patients were recruited between September 2016 and October 2017 and provided written informed consent following a minimum 24‐hour period of consideration. The patient inclusion criteria were 1) men referred for prostate mp‐MRI following previous biopsy more than 6 months earlier, and 2) biopsy‐naive men presenting a clinical suspicion of prostate cancer. Patient exclusion criteria included 1) men unable to have an MRI scan, or in whom artifact would reduce the quality of the MRI, 2) men unable to give informed consent, 3) previous treatment (prostatectomy, radiotherapy, brachytherapy) of prostate cancer, 4) ongoing hormonal treatment for prostate cancer, and 5) previous biopsy within 6 months of scheduled mp‐MRI. Five subjects were excluded throughout the course of this study, four due to MR contraindications and one due to a technical fault. In this study an assessment was made on a broad range of subjects, including men for whom it was decided a biopsy was not necessary.

### *MRI Acquisitions*

Subjects were scanned on a 3.0 T scanner (Philips Achieva; Philips Medical Systems, Best, The Netherlands) using a 32‐channel cardiac coil. A multiecho spin‐echo sequence with an echo spacing of 31.25 msec and repetition time (TR) of 8956 msec was used. The other parameters were: NE = 32; field of view (FOV) = 180 × 180 mm; acquired voxel size = 2 × 2 × 4 mm; scan duration = 5 minutes 50 seconds. DWI was acquired for VERDICT fitting with single diffusion encoding (SDE) single‐shot EPI sequences over six b‐values (0, 90, 500, 1500, 2000, 3000 s/mm^2^). TR/TE = 2000–3707/50–80 msec; FOV = 220 × 220 mm; voxel size = 1.3 × 1.3 × 5 mm; scan duration = 12:57.^16^ A standard mp‐MRI protocol was also conducted on these patients, as detailed in previous work.^17^

### *Regions of Interest (ROIs) and Histologic Examination*

A board‐certified radiologist with 5 years of experience in prostate mp‐MRI reporting (F.G.) contoured 97 areas of either malignant or benign tissue. The lesions had previously been located in the mp‐MRI images and the primary lesions were then located and contoured in a single slice of the 93.75 msec echo of the ME‐T_2_ image with no knowledge of the LWI maps. In the case of malignant tissue the entire lesion was outlined, whereas in healthy tissue the ROI from the lesion was copied into a region of healthy tissue in the same prostate zone. The 93.75 msec echo was chosen for its similar echo time to a traditional axial T_2_ weighted prostate image (~100 msec). In all, 98 ROIs were also contoured on the corresponding slice of the ADC maps. These ROIs were drawn to directly correspond to those contoured on the ME‐T_2_ images. The number of ROIs were slightly different in the ADC and ME‐T_2_ images due to two ME‐T_2_ images having not been correctly acquired and one ADC image having a large artifact in the ROI. Each area of benign tissue or lesion was assigned a PI‐RADS v2 score based on the standard mp‐MRI images, acquired in addition to the VERDICT and ME‐T_2_ images. PI‐RADS v2 is a method of scoring tissue on a scale of 1–5, with 1 meaning clinically significant cancer is highly unlikely to be present and 5 meaning clinically significant cancer is highly likely to be present. In the peripheral zone (PZ) of the prostate, where the majority of tumors arise, this scoring is primarily informed by diffusion images, with axial T_2_ and DCE images used when the diffusion image is indeterminate. Histological grading using a targeted transperineal template biopsy was available on a subset of 31 of the ROIs. The Gleason grading system was used to evaluate the biopsy tissue samples.^18^ Our primary analysis uses ROIs drawn without knowledge of the LWF maps to avoid bias. If LWF maps are used directly in the future, an indication of reproducibility between readers will be informative. In a substudy of 16 patient datasets, two separate readers each drew 16 ROIs on the LWF maps themselves and the median parameter values were evaluated using a correlation and Bland–Altman analysis.

### *Data Processing*

Sabouri et al^14^ used a regularized nonnegative least squares (NNLS) algorithm to fit a multiexponential model. The NNLS algorithm fits a large number of exponentials (>100) to the signal decay curve, including a regularizing term in the error minimization to compensate for problems associated with having a large number of unknown variables compared with the number of echoes. A large number of exponentials also seems inappropriate because in previous works,^13^, ^14^, ^15^ only two compartments were usually identified in the prostate gland.

Hence, for this work we have constrained the model to two compartments, each with a Gaussian probability distribution of T_2_ values. The choice of Gaussians is a mathematically simple choice and, based on preliminary analysis, the difference between Gaussian, log‐Gaussian, and gamma distributions made little difference to the overall fit of the signal decay. Hence a two‐Gaussian model was fitted to the individual T_2_ signal decay curves using a least‐squares regression. These two Gaussian distributions model the tissue as a combination of a luminal compartment with long T_2_ values and a compartment composed of stroma and epithelia with shorter T_2_ values. The probability density *p* over T_2_ value *T*_*2*_ in a particular pixel is given by:

(1) $$p\left(T2\right)=\frac{\alpha }{\sigma_{1}\sqrt{2\pi }}\exp\left(-\frac{{\left(T2-\mu_{1}\right)}^{2}}{2\sigma_{1}^{2}}\right)+\frac{1-\alpha }{\sigma_{2}\sqrt{2\pi }}\exp\left(-\frac{{\left(T2-\mu_{2}\right)}^{2}}{2\sigma_{2}^{2}}\right)$$

with the signal intensity *S* at each echo time *TE* subsequently calculated as:

(2) $$S=M_{0}\int_{0}^{\infty }p\left(T2\right).\exp\left(-\frac{\mathrm{TE}}{T2}\right)\mathrm{dT}2$$

This model fitting minimizes the mean squared error between the actual signal and the modeled signal, using the Levenberg–Marquardt algorithm to optimize over six parameters: the absolute signal magnitude (*M*_*0*_), the magnitude ratio between the two peaks (α), the means of the two components (μ_1_ and μ_2_), and the variances of the two components (σ_1_ and σ_2_). The values of μ_1_ and μ_2_ were constrained to be 0–200 msec and 200–3000 msec, respectively; none of the other parameters were constrained. The starting values for the constrained model for *M*_*0*_, μ_1_, and μ_2_ were calculated once for each subject by taking an average intensity for each echo over all pixels in the prostate and carrying out a biexponential fit on the averaged signal decay across the ROI. This study used the short and long T_2_ values of the biexponential fit as initial estimates for μ_1_ and μ_2_ and the mean of the magnitudes of each exponential as the initial estimate for *M*_*0*_. Initial values of σ_1_ and σ_2_ were set at 5 × 10^‐4^, which was in the range of the standard deviation of the NNLS peaks in previous LWI fittings. This reduction in the number of parameters compared with the unconstrained model should provide a more reliable fit and operate using fewer data points, permitting echo trains with fewer echo signals.

The areas under the individual peaks, *A*_*1*_ for the shorter T_2_ peak and *A*_*2*_ for the longer T_2_ peak, were calculated by integrating the respective Gaussians using their magnitude, mean, and variance. The LWF was then calculated as the fraction of the total area under the distribution curve attributed to the peak with the longer T_2_:

(3) $$\mathrm{LWF}=A_{2}/\left(A_{1}+A_{2}\right)$$

For each of these parameters a map was created across the entire prostate. Then the ROIs produced earlier on the 93.75 msec echo of the ME‐T_2_ image were superimposed onto these parameter maps and the median values of these parameters were calculated for each ROI. All data were processed using MatLab (MATLAB and Statistics Toolbox Release 2017a, MathWorks, Natick, MA).

The VERDICT model, when applied to the prostate, is a three‐compartment diffusion‐based microstructural model that characterizes water diffusion into vascular, intracellular (IC), and extracellular‐extravascular space (EES) compartments. The IC compartment has volume fraction (fIC), diffusivity (dIC), and cell radius (R) as parameters. The EES compartment has volume fraction (fEES) and diffusivity (dEES) as parameters. The vascular model has volume fraction (fVASC) and pseudo‐diffusivity (P) as parameters.

### *Statistics*

In order to create a 95% confidence interval for the simulation data, bias corrected and accelerated percentile bootstrapping was used on 1000 bootstrap samples.

Differences were characterized between the median parameter values of ROIs with different PI‐RADS v2 groupings of scores and determined using a logistic regression model combined with 5‐fold crossvalidation. Three comparisons were made in this way. The comparison between the scores PI‐RADS 1,2 vs. PI‐RADS 3,4,5 aims to divide those lesions needing further action from those that do not. The other two comparisons, PI‐RADS 1,2 vs. PI‐RADS 3, and PI‐RADS 3 vs. PI‐RADS 4,5, both aim to investigate the model's ability to discern between the three main categories of negative (1,2), indeterminate (3), and positive (4,5) disease. *P* < 0.05 was taken to be significant. The mean values for sensitivity, specificity, and area‐under‐curve (AUC) values across the five‐folds were also computed using a receiver operating characteristic (ROC) analysis. Sensitivity and specificity values were calculated from the ROC analysis using an operating point with the shortest distance to the point of perfect discrimination. A logistic regression was performed on the median values of those ROIs with a corresponding histological grading in order to discern malignant (Gleason 3 + 3 and above) from nonmalignant tissue.

To detect significant statistical differences between the values of AUC for LWF and ADC when predicting PI‐RADS v2 categories, a Kruskal–Wallis nonparametric statistical test was performed on the AUC values of each of the five‐folds of the crossvalidation. To detect significant statistical differences between the values of AUC for LWF and ADC when predicting Gleason categories, a Kruskal–Wallis nonparametric statistical test was performed on 1000 bootstrapped examples. For both the PI‐RADS v2 and Gleason Score Kruskal–Wallis tests, *P* < 0.05 would suggest with 95% confidence that the null hypothesis (that the AUC values of ADC and LWF come from the same distribution) be rejected.

In order to assess the relationship between LWI and the VERDICT diffusion model for prostate,^16^ a Pearson's correlation coefficient was calculated between the VERDICT fIC, fEES, fVASC parameters and each of the parameters of LWI separately. The fIC parameter is of particular interest due to the fact that it has previously shown significant difference between PCa and normal tissue^18^ and that it represents the cellular compartment within the tissue, and hence might be expected to negatively correlate with the LWF.

A standard Bland–Altman analysis was carried out on the median values within the subset of 16 ROIs to analyze the reproducibility of this method.

## Results

The age range of the 65 patients was 49–79 years with a mean of 65. Of the T_2_ ROIs with PI‐RADS v2 scoring, there were 31 PI‐RADS 1, 32 PI‐RADS 2, 18 PI‐RADS 3, 5 PI‐RADS 4, and 11 PI‐RADS 5. Similarly for the diffusion ROIs, there were 30 PI‐RADS 1, 32 PI‐RADS 2, 18 PI‐RADS 3, 7 PI‐RADS 4, and 11 PI‐RADS 5. Of the histologically examined ROIs, 16 were found to be benign, three were Gleason 3 + 3, six were 3 + 4, three were 4 + 3, two were 4 + 4, and one was 4 + 5.

The simulation results in Table 1 show that the proposed two‐Gaussian method with 32 echoes has a similar accuracy to the original LWI at determining the LWF over a range of ground truth tissue models.

**Table 1:** Mean Estimated LWF Values for Both the Constrained and Unconstrained Models Using Different Ground Truth LWF Values in Simulation

		Ground truth LWF		
Pulse sequence	Model fitting	0	0.1	0.2
32‐echo & 31.25 msec echo spacing	Two‐Gaussian	0.0074 (0.0019,0.0747)	0.1001 (0.0918,0.1174)	0.1913 (0.1817,0.2605)
32‐echo & 31.25 msec echo spacing	NNLS	0.0003 (0.0002,0.0005)	0.0867 (0.0827,0.0900)	0.1802 (0.1691,0.1884)
64‐echo & 25 msec echo spacing	Two‐Gaussian	0.0008 (0.0004,0.0104)	0.0976 (0.0914,0.1042)	0.1996 (0.1905,0.2203)
64‐echo & 25 msec echo spacing	NNLS	0.0003 (0.0002,0.0040)	0.0900 (0.0859,0.0957)	0.1829 (0.1698,0.1885)

The mean values using both the delta and Gaussian ground truth models over a range of μ_1_ and μ_2_ values. In brackets are the 95% confidence interval bounds.

Table 2 shows the results of the statistical tests for in vivo PI‐RADS v2 score groupings.

**Table 2:** Three Separate Analyses Using LWF and ADC to Predict PI‐RADS v2 Scores

Scores	1,2v3				3v4,5				1,2v3,4,5			
Test	*P‐*val.	AUC	Sens.	Spec.	*P‐*val.	AUC	Sens.	Spec.	*P‐*val.	AUC	Sens.	Spec.
LWF	0.0047	0.7857	63.3	84.0	0.0129	0.8667	80.0	76.7	0.0001	0.8809	80.6	78.7
ADC	0.3947	0.6546	48.7	80.9	0.0805	0.7467	73.3	79.3	0.0346	0.6909	59.4	77.4

The P‐value comes from the logistic regression model and the other statistics are from an ROC analysis.

Table 3 presents the comparison of the proposed method with histologically confirmed malignant lesions and shows values of 0.81 and 0.75 (AUC), 75% and 83% (sensitivity), and 87% and 67% (specificity), respectively, for LWF and ADC.

**Table 3:** ROC Analysis of LWF in Detecting Malignant Lesions (Gleason 3 + 3 and Above)

Variable	AUC	Sensitivity	Specificity
LWF	0.81	75%	87%
ADC	0.75	83%	67%

Table 4 shows the results of Kruskal–Wallis tests designed to test whether there is a significant difference between the AUC values produced by ADC and those produced by LWF. These tests were performed on each of the four score groupings investigated. In none of the comparisons made can the results prove with 95% confidence that the ADC and LWF are producing significantly different AUC values.

**Table 4:** *P‐*values of Kruskal‐Wallis Tests Between ADC and LWF for the ROC Analyses Performed on Each of the Four Score Groupings Tested

Score groupings	*P‐*value
PI‐RADS 1,2 v 3	0.0758
PI‐RADS 3 v 4,5	0.1246
PI‐RADS 1,2 v 3,4,5	0.0758
Gleason 3 + 3 and above	0.7771

A P‐value of 0.05 means that the null hypothesis, that the ADC and LWF predictions have the same AUC values, can be rejected with 95% confidence.

Table 5 shows the correlations between the individual parameters of the LWI model and three parameters from the VERDICT diffusion model. With respect to fIC, significant positive correlations greater than 0.5 were seen for α and *A*_*1*_ and significant negative correlations less than –0.5 were seen for *A*_*2*_ and LWF. With respect to fEES, significant positive correlations greater than 0.5 were seen for *A*_*2*_ and LWF and significant negative correlations less than –0.5 were seen for α. None of the LWI parameters show a correlation greater than 0.5 or less than –0.5 with fVASC.

**Table 5:** Correlation Between LWI Parameters and the Intracellular Fraction (fIC), Extracellular‐Extravascular Fraction (fEES), and Vascular Fraction (fVASC) Parameters of the VERDICT Diffusion Model

		M_0_	*α*	*μ*_1_	*μ*_2_	*σ*_1_	*σ*_2_	A_1_	A_2_	LWF
fIC	Corr.	0.3935	0.6197	–0.1313	0.2518	0.0253	0.2715	0.516	–0.6017	–0.6184
	*P‐*value	0.0003	0.0000	0.2458	0.0242	0.8237	0.0148	0.0000	0.0000	0.0000
fEES	Corr.	–0.2319	–0.5411	0.0690	–0.2735	0.0576	–0.2612	–0.3635	0.5825	0.5644
	*P‐*value	0.0385	0.0000	0.5429	0.0141	0.6121	0.0193	0.0009	0.0000	0.0000
fVASC	Corr.	–0.1700	–0.0812	0.0591	0.0650	–0.0965	–0.0041	–0.1618	0.0024	0.0384
	*P‐*value	0.1317	0.4739	0.6026	0.5670	0.3945	0.9711	0.1515	0.9834	0.7355

**Figure 2:**
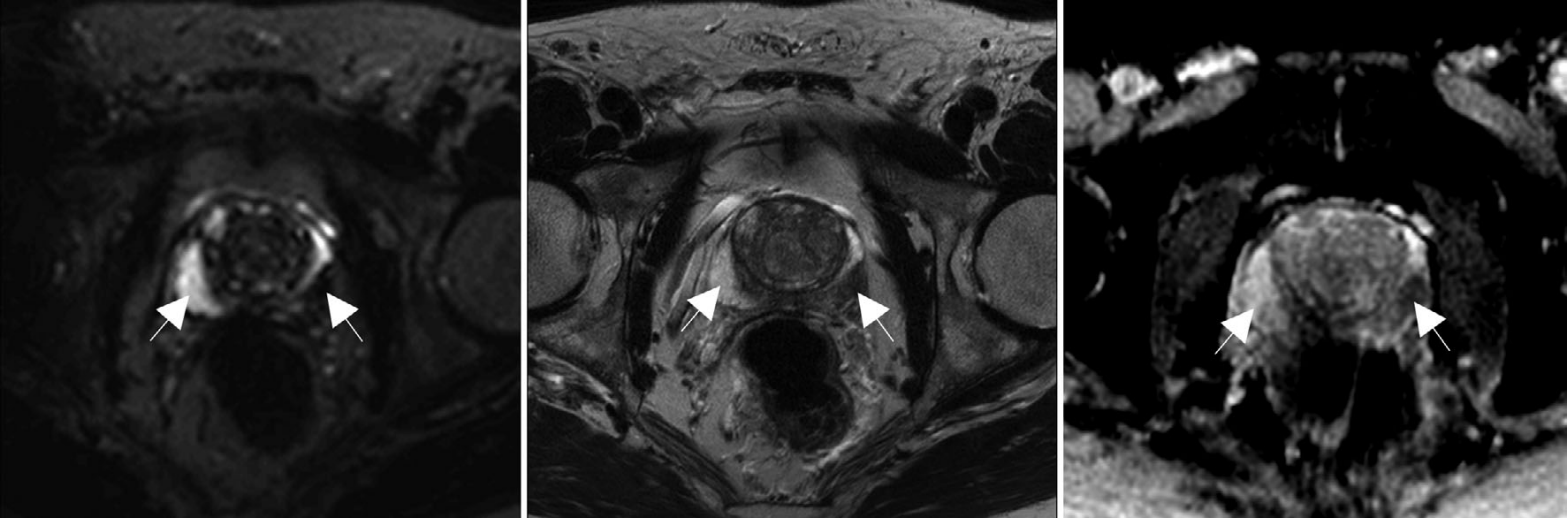
LWF map, axial T_2_ image, and ADC map for one patient. The region of healthy tissue is highlighted by the arrow on the left of each image, the tumor by the arrow on the right of each image. This figure shows distortions in the PZ in the diffusion‐weighted image, highlighting a disadvantage of DWI over ME‐T_2_ modeling.

Figure 2 shows the LWF map for one patient alongside the axial‐T_2_ and ADC maps from the same subject. Note the higher LWF in the PZ, consistent with histological findings of large regular glandular lumen and loosely woven stroma in the PZ.

**Figure 3:**
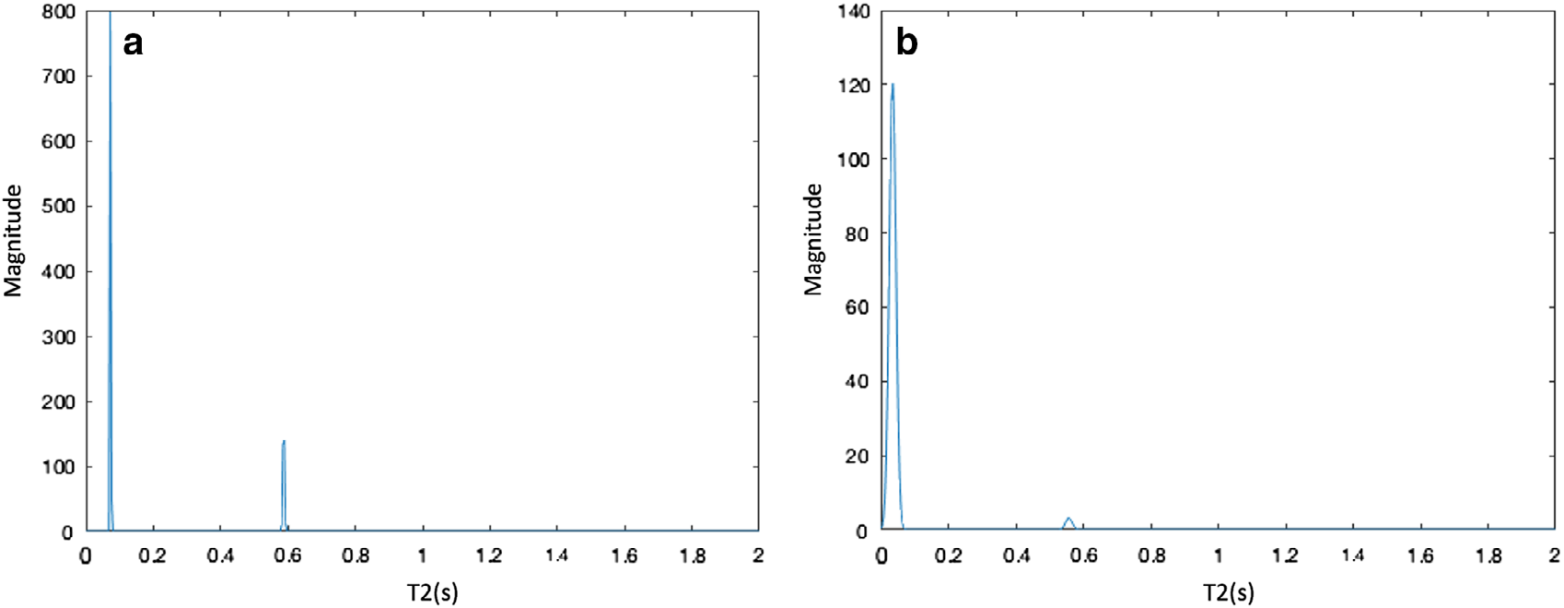
T_2_ distributions for an example pixel in **(a)** normal tissue and **(b)** tumor. These pixels are taken from the subject in Fig. 2 in the regions highlighted by the arrows.

Figure 3 shows graphs for the T_2_ distributions from single example voxels in healthy and cancerous tissue, respectively. Clear changes in the distributions are visible.

The Bland–Altman analysis of the median LWF values of the subset of 16 ROIs produced an r^2^ value of 0.928, a bias of 0.013, and limits of agreement of –0.151 and 0.124, with a mean value of 0.179.

## Discussion

The main aim of this work was to compare a simplified LWI with ADC for the prediction of PI‐RADS v2 scores. The LWF predictions showed similar AUC values to ADC, suggesting that LWF is able to discriminate between clinically relevant PI‐RADS v2 groupings of scores as well as ADC. Given that the PI‐RADS v2 scoring scheme uses ADC as a major component and might thus be expected to favor ADC, it is interesting to find that LWI alone can predict PI‐RADS v2 score as well as ADC alone. The ability of LWF to differentiate tumor from normal tissue using histologically examined ROIs further reinforces the idea that multiecho T_2_ modeling shows promise as a method for detecting and grading PCa.

Our results suggest that LWI may be a useful tool in PCa detection. If using the LWF measure in the future, ROIs may be drawn directly on LWF maps, in which case the repeatability between readers is an important consideration. The correlation and Bland–Altman analysis carried out on a subset of our patients provides an indication of the expected variability. A more extensive quantification of the value of LWI in PCa detection requires larger and multicenter studies and the results presented here can inform those studies.

The correlations between LWI and the VERDICT model are intended to give an insight into how LWI relates to tissue microstructure. An increase in LWF represents an increase in the fractional volume of luminal space within the prostate. As PCa typically invades the luminal space and reduces the luminal fractional volume, a reduced LWF value is expected within a tumor. The parameters with significant correlations greater than 0.5 in magnitude suggest that as the volume fraction of the luminal space decreases the volume fraction of the intracellular compartment increases and the volume fraction of the extracellular‐extravascular compartment decreases.^16^ These correlations with the VERDICT model parameters suggest that LWI is sensitive to the underlying tissue microstructure.

MRI is utilized in the prostate for the detection and staging of tumors. LWI allows for the collection of microstructural information without the distortion artifacts seen in diffusion imaging. In the future, it might be that either less diffusion data need to be acquired, or that complementary information from LWI improves the efficacy of mp‐MRI given the different tissue compartments that they are designed to probe. Further sequence optimization to increase coverage of the prostate and reducing the thickness of the slices would be beneficial prior to testing on a much larger number of subjects and across multiple centers. A future prospective assessment should also include a multiple reader study to quantify the variability introduced by radiological placement of ROIs.

Although a PI‐RADS score can be allocated to any lesion, a limitation of this study is that not all patients subsequently received biopsy, limiting the ability to perform full histological correlations. Another limitation is that biopsy is prone to sampling error, which can lead to the undergrading of tumors.^19^ Sampling error could be reduced by using whole‐mount histopathology but this would lead to a bias in the Gleason grades of the lesions studied due to the fact that radical prostatectomies are only carried out on subjects with more significant lesions. Furthermore, the subjects in this study were a subset of a larger prospective study,^17^ meaning that the analysis done in this article was retrospective. A larger number of subjects could prove more conclusively the hypothesis that LWI performs at least as well as ADC in discerning the PI‐RADS v2 score and Gleason score. This would allow for a reduced number of protocols for grading prostate cancer, reducing overall scan times. The AUC for the detection of PCa is also lower in this study than previous results,^15^ possibly due to previous studies using patients scheduled for retropubic prostatectomy, weighting the lesions towards more advanced tumors.

In conclusion, this work suggests that LWI is sensitive to the tissue microstructure and can be as effective as ADC in the classification of lesions using the PI‐RADS V2 scores while providing images with minimal distortions.
